# Formulation of Bacteriophage for Inhalation to Treat Multidrug-Resistant Pulmonary Infections

**DOI:** 10.14356/kona.2025016

**Published:** 2024-08-14

**Authors:** Vaibhav Pathak, Hak-Kim Chan, Qi Tony Zhou

**Affiliations:** 1 Department of Industrial and Molecular Pharmaceutics, College of Pharmacy, Purdue University, USA; 2 Advanced Drug Delivery Group, School of Pharmacy, Faculty of Medicine and Health, The University of Sydney, Australia

**Keywords:** bacteriophage therapy, dry powder inhalers, antimicrobial resistance, pulmonary infection

## Abstract

Rapid development of antibiotic resistance in pathogenic bacteria and a decline in the pharmaceutical development of new antibiotics are pushing the research community to explore alternative antimicrobials that can replace or complement antibiotics. Bacteriophages (or, phages) are naturally occurring viruses that can kill bacteria with high specificity and can evolve to target resistant bacteria. Phages have been historically employed as antimicrobial agents, but they were overshadowed by the emergence of antibiotics. With a renewed focus on phages, it is important to study their clinical efficacy, safety, and formulation. Pulmonary infections have a large burden of global morbidity and frequently involve multidrug-resistant pathogens such as *Acinetobacter baumannii*, *Klebsiella pneumoniae*, *Mycobacterium tuberculosis*, and *Pseudomonas aeruginosa*. Therefore, this can be an important area of application of phages. Dry powder inhalers can be an effective strategy to deliver phages to the lungs because they are easy-to-use, portable, and capable of delivering a higher lung dose than oral or intravenous route. They also have longer shelf life and lower cold storage requirements than solutions. Therefore, the aim of the current review is to summarize recent findings on bacteriophage dry powder formulations, particularly focusing on the effect of various excipients and manufacturing factors on phage titer preservation.

## Graphical Abstract

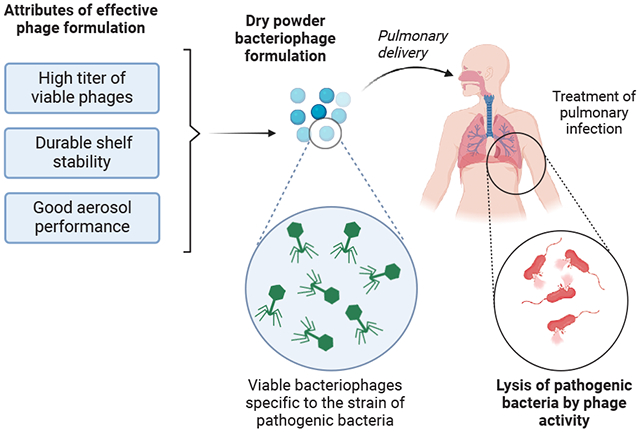

## 1. Introduction

Gram-negative bacteria like *Acinetobacter baumannii*, *Klebsiella pneumoniae*, and *Pseudomonas aeruginosa* are a serious threat to human health due to their ability to develop resistance to a broad range of antibiotics (Livermore, 2003). For instance, a review by Livermore in 2002 reported that 1 % of *P. aeruginosa* isolates in the USA showed resistance against six of the most relevant antibiotics: amikacin, ceftazidime, ciprofloxacin, gentamicin, carbapenems, and piperacillin (Livermore, 2002). In 2014, 51,000 cases of healthcare-associated *P. aeruginosa* infections were reported in the USA, and 13 % were multidrug-resistant (Rossolini et al., 2014).

The problem of antimicrobial resistance is intensified by a steadily decreasing number of new antibiotics under development against Gram-negative pathogens over the last three decades (Ventola, 2015). Developing antibiotics for Gram-negative bacteria has become less profitable for the pharmaceutical industry because of the shorter duration of use (Bartlett et al., 2013), cheaper prices, and unpredictable risk of resistance development that can curtail sales (Gould and Bal, 2013). Given this shortage of new classes of antibiotics, researchers and clinicians have turned to exploring alternative antimicrobial strategies like bacteriophage, antimicrobial peptides, quorum sensing inhibitors, biofilm inhibitors, and antibiotic adjuvants (Scoffone et al., 2024).

Bacteriophages are naturally occurring viruses that infect bacteria. Bacteriophages that are obligately *lytic* infect bacterial cells for self-replication and release progeny through cell lysis. Progeny phages can infect other bacterial cells. Lytic phages can therefore be effective antibacterial agents. Phages have not yet been widely adopted as a treatment option in Western countries, but they can be a potential treatment due to several unique characteristics: specificity to bacteria, ability to self-propagate in the presence of bacteria, and ability to co-evolve with bacteria (Loc-Carrillo and Abedon, 2011).

Respiratory infections are one of the leading causes of mortality. Chronic and refractory lung infections often occur in patients with compromised lungs, such as those with chronic obstructive pulmonary disorder (COPD) and cystic fibrosis (CF) (Hauser et al., 2011). The treatment of chronic lung infections can be complicated by inefficient drug delivery to the lungs, the development of multidrug resistance in chronic cases, and a shortage of new antibiotics and alternative antimicrobial drugs. Phage therapy can be an alternative strategy for treating lung infections resistant to antibiotics.

Phage therapy has several advantages over traditional antibiotics in terms of their ability to treat bacterial infections (Kortright et al., 2019): (1) Phages have a high host specificity, which reduces off-target effects on the natural microbiome of the patient. Moreover, phages do not infect human cells; (2) Because phages can self-replicate via the lytic infection of bacteria, the dose of the phage can be amplified in the presence of the host bacteria. This indicates that phages can be used at lower doses than antibiotics; (3) Phages are naturally capable of evolving to overcome the phage resistance mechanisms of bacteria. Thus, unlike antibiotics, iterations of a phage can be employed to target resistance bacteria; (4) Chronic lung infections often develop biofilms that are practically impermeable to antibiotics. Phages can penetrate biofilms (Chan et al., 2018; Loc-Carrillo and Abedon, 2011), making them highly effective against chronic bacterial infections.

## 2. Efficacy of phage therapy

Phage therapy for lung infections has shown promise in animal studies and clinical cases. Additionally, phage therapy has often been used for the treatment of bacterial infections in eastern European countries for decades (Abedon, 2015). Some recent studies are summarized below.

### 2.1 Phage testing in animal models

Debarbieux et al. (2010) tested the effect of intranasal phage administration in acute lung infection models using bioluminescent *P. aeruginosa* (PAK lumi). The live bacteria population in mice was estimated by quantifying photon emission. Bacteriophage (PAK-P1) pretreatment of mice 24 h before introduction of bacterial infection using phages at a multiplicity of infection (phage-to-bacteria ratio) of 10 resulted in 5 times lower photon emission after 2 h than phosphate buffer saline control pretreatment. All phage-pretreated mice survived for 16 days, whereas all mice in the control group died within 2 days (Debarbieux et al., 2010). This demonstrates the great potential of preventive phage therapy against the known risk of bacterial challenge. Semler et al. (2014) showed that aerosolized phage delivery (Myoviridae KS12) to mice lungs resulted in a 2-log reduction in the median bacterial load of acute *Burkholderia cenocepacia* (K56–2, highly antibiotic-resistant) infection in mice. Yang et al. (2015) demonstrated complete eradication of *P. aeruginosa* D9 cells in a murine hemorrhagic pneumonia model and 100% survival of mice using a single dose of an N4-like phage (YH6) with an MOI of 0.1 administered intranasally 2 h after infection. Cao et al. (2015) showed that when murine pneumonia induced by the multi-resistant *Klebsiella pneumoniae* strain 1513 was treated intranasally with phages at an MOI of 10, 80 % of the mice survived. In comparison, none of the control-treated mice survived. Phage-treated mice exhibited reduced bacterial cell burden, lower loss of body weight, and exhibited lower levels of inflammatory cytokines in the lungs (Cao et al., 2015).

### 2.2 Clinical application of phages

Hoyle et al. (2018) reported the phage treatment of a cystic fibrosis (CF) patient with a multidrug-resistant *Achromobacter xylosoxidans* lung infection. A cocktail of two *Achromobacter* bacteriophages (*Siphoviridae* family) (6 × 10^8^ PFU dose) was nebulized for inhalation and orally administered twice daily for 20 days. The course was repeated four times over a year. The patient’s lung function improved significantly, dyspnea resolved, and cough reduced (Hoyle et al., 2018). Other clinical and animal studies on bacteriophage treatment have been extensively reviewed by Abedon (2015) and Bhadoriya et al. (2023).

By employing two or more independent modes of bacterial killing, bacterial killing can be improved and the chance of selecting resistant mutants can be reduced (Torres-Barceló and Hochberg, 2016). Phage cocktails containing two or more phages specific to a pathogen can improve bacterial killing and prevent development of resistance. Similarly, the combination of phages and antibiotics has also shown promise (Tagliaferri et al., 2019). Some phage-antibiotics combinations can show synergistic antibacterial action (Comeau et al., 2007; Kamal and Dennis, 2015; Ryan et al., 2012; Uchiyama et al., 2018). Ruest et al. (2023) demonstrated synergistic bacterial killing in some phage-phage and phage-colistin combinations against *B. cenocepacia*. The study also observed “phage steering”, where phage resistance can be exploited to re-sensitize bacteria to antibiotics. Some cases of phage-induced resistance in *Burkholderia cenocepacia* altered the structure of its lipopolysaccharide membrane, making it susceptible to the action of immune components in serum and antibiotics that target the membrane, such as colistin (Ruest et al., 2023).

In addition to the clinical use of phage therapies in East Europe, several phage therapies are currently under development in the USA and Europe: BX004 phage by BiomX is under clinical evaluation for treating multi-resistant *P. aeruginosa* involved in cystic fibrosis (ClinicalTrials.gov ID NCT05498363). Armata Pharmaceuticals is currently studying the safety, kinetics, and efficacy of the inhaled AP-PA02 phage in subjects with non-cystic fibrosis bronchiectasis and chronic pulmonary *P. aeruginosa* infection (ID NCT05616221). Table 1 lists bacteriophage products currently under development.

### 3. Powder formulation of bacteriophages for inhalation

Successful phage treatment depends on the delivery of a high phage titer to the infection site. Pulmonary delivery of a wide variety of drugs shows higher target site deposition and thus requires a lower dose than parenteral or oral delivery.

Nebulization, pressurized metered-dose inhalers (pMDIs), and dry powder inhalers are the most common pulmonary drug delivery methods. Dry powder inhalers stand out as a dosage form for inhaled delivery because they are portable and easy to use by patients (Kwok and Chan, 2014; Zhou et al., 2014). It can deliver a higher dose of the drug substance to the lungs in a shorter time than nebulizers and pMDIs. Importantly, dry powders tend to be physically and chemically more stable than solutions or suspensions (Ke et al., 2022). It is especially beneficial to formulate labile biological drugs like proteins, viruses, and monoclonal antibodies as dry powders because of their improved storage stability (Santana et al., 2014; Walters et al., 2014). Therefore, this study focused on the formulation of bacteriophages as inhaled dry powders.

**Fig. 1:**
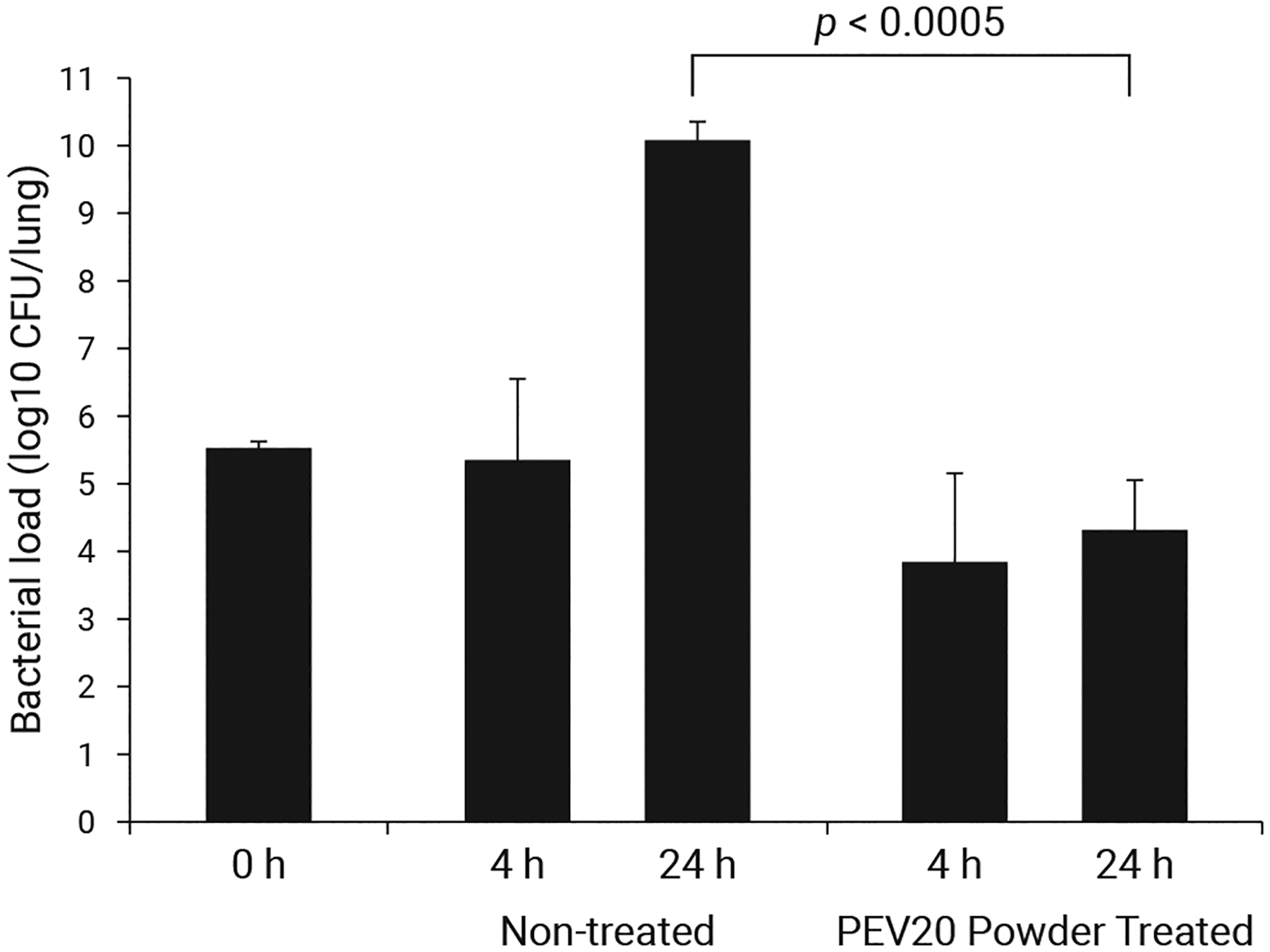
Comparison of PEV20 phage dry powder treatment (1.4 × 10^7^ to 1.2 × 10^8^ PFU/lung) and no treatment on the bacterial load of *Pseudomonas aeruginosa* FADDI-PA001 in a mouse lung infection model at 4 and 24 h after infection, reprinted with permission from Ref. (Chang et al., 2018). Copyright (2018) American Society for Microbiology.

Chang et al. (2018) conducted a proof-of-principle study to investigate the *in vivo* effects of phage dry powders on *Pseudomonas aeruginosa* infection (Fig. 1). The lungs of neutropenic mice were infected using a suspension of FADDI-PA001 bacteria via intratracheal instillation, which resulted in an average load of 3.4 × 10^5^ CFU/lung. Phage powders were administered intratracheally at a dose of 1.4 × 10^7^ to 1.2 × 10^8^ PFU/lung. After 24 h, the phage-treated mice had a 5.3 log_10_ lower bacterial count than the untreated mice (Chang et al., 2018).

Dry powders for inhalation are often engineered to have appropriate aerodynamic particle size and physicochemical properties that facilitate their efficient delivery to the lungs (Hoppentocht, 2016; Ke et al., 2022). These powders should resist physical changes that can occur due to moisture sorption and agglomeration during storage because it affects their dispersion efficiency. Furthermore, the formulation of biological drugs as dry powders presents additional requirements. Biologic drug substances, including phages, are highly susceptible to loss of therapeutic activity due to external stresses. It is important to design a manufacturing method and select formulation components that minimize phage inactivation during processing and storage.

**Fig. 2:**
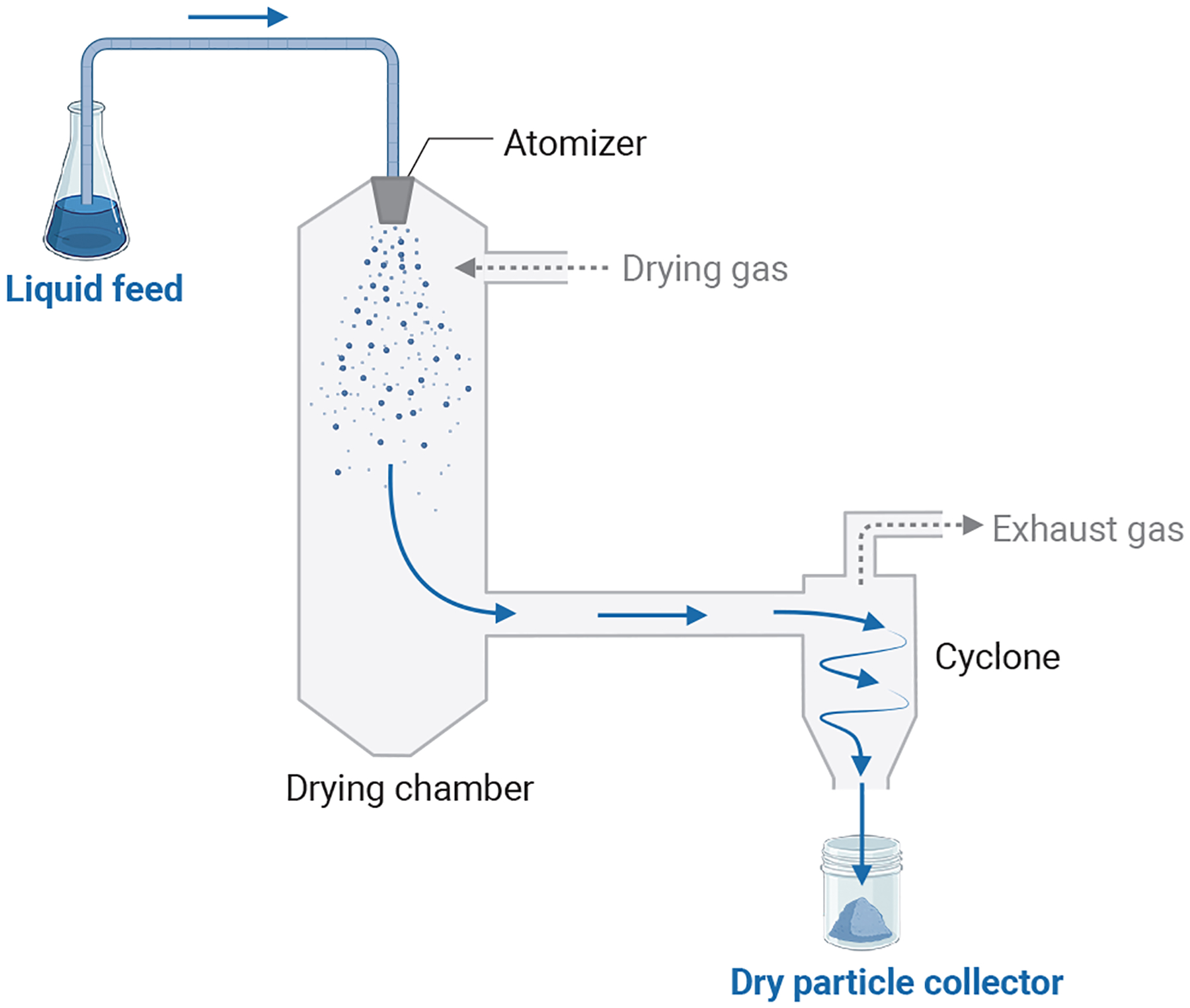
Schematic of the spray drying process. Created on BioRender.com.

**Fig. 3:**
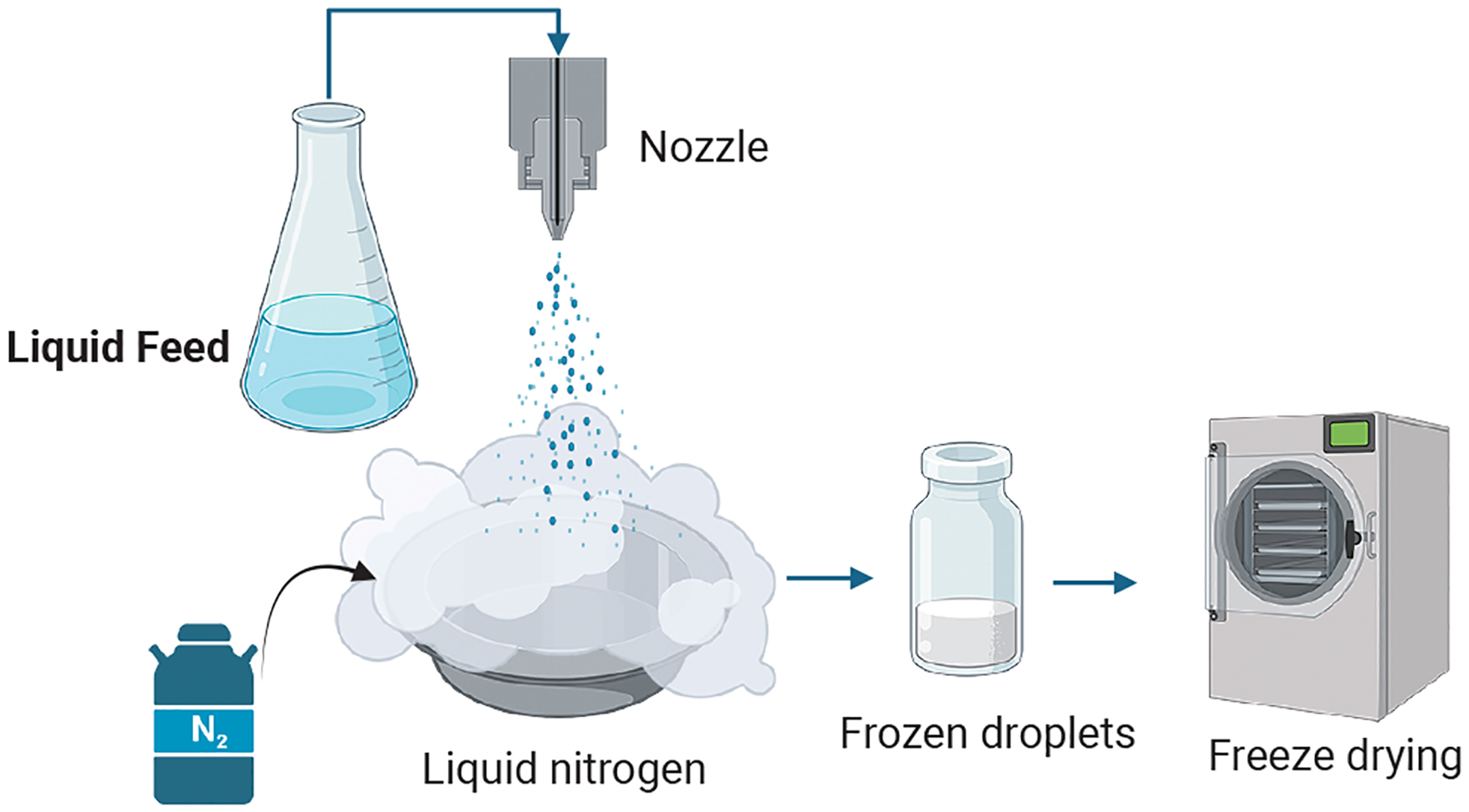
Schematic of spray freeze drying. Created on BioRender.com.

Dry powders for inhalation are commonly prepared by spray drying, lyophilization-micronization, spray freeze drying, and blending with lactose carriers. The requirement for higher antimicrobial drug doses limits the use of lactose carrier blends because of the high powder burden (Patton and Byron, 2007). Spray drying is a particle engineering technology involving hot air flow to dry an atomized solution or suspension (Fig. 2). Lyophilization is the process of freezing and removing moisture from a drug solution at low temperature and pressure via sublimation. Lyophilized dry cake can be micronized to generate inhaled dry powder (Golshahi et al., 2011). Spray freeze drying involves vacuum or atmospheric drying of frozen atomized drug solution droplets generated by rapid exposure to liquid nitrogen or other cryogens (Fig. 3).

Several studies have investigated the preparation of dry powder phage formulations using spray drying, wherein they study the effects of manufacturing parameters and excipients on the formulation stability and aerosol performance. During spray drying, the formulation is exposed to shear, thermal, and desiccation stresses (Leung et al., 2016; Matinkhoo et al., 2011; Vandenheuvel et al., 2013). These stresses can induce denaturation of the capsid protein, breakage of the tail, or loss of enclosed nucleic acid material, which can cause the phage to lose its ability to infect host bacteria.

Sugars like trehalose, lactose, and sucrose are commonly used as lyoprotectants in protein formulations because they prevent protein denaturation in the absence of water. Sugars are believed to have glass-forming ability that can immobilize proteins in their native conformations (Chang and Pikal, 2009). Another mechanism of stabilization attributed to sugars is their ability to form hydrogen bonds with proteins (Grasmeijer et al., 2013). In this way, sugars replace hydrogen-bonding interactions between proteins and water. It is possible for one or both mechanisms to be responsible for protein stabilization. Because a phage body is essentially a complex assembly of proteins enclosing nucleic acids, it can also be stabilized in the dry state using sugars. Several studies have investigated the ability of these sugars to stabilize phages during drying and subsequent storage. Indeed, phages were shown to be stable when the glass transition temperature of the sugar matrix was 46 °C above the storage temperature, which supports the role of immobilization by the glassy matrix in phage stabilization (Chang et al., 2020).

There are limited studies on the formulation of bacteriophages as dry powders for inhalation. These studies selected diverse phages that target multidrug-resistant strains of pathogenic bacteria like *Pseudomonas aeruginosa*, *Burkhoderia* complexes, and *Acinetobacter baumannii*. Most of these studies focused on spray drying to manufacture inhalable phage formulations (Chang et al., 2017; Leung et al., 2017; Matinkhoo et al., 2011). Other studies have explored spray freeze drying and a combination process of lyophilization-milling (Golshahi et al., 2011; Leung et al., 2016). The main objective of these studies was to determine processing conditions and excipient combinations that minimized the loss of phage viability during preparation and/or storage.

The number of viable phages is generally quantified using a double-agar plaque assay, which is considered the gold standard biological assay and is based on the interaction of a phage with its host bacteria (Ács et al., 2020). A phage sample is suspended and serially diluted. These diluted suspensions are poured over a lawn of actively growing host bacteria and incubated. The active phages in the solution infect the bacteria and begin the lytic cycle of phage propagation. Over time, bacterial cell lysis can cause visible plaques to appear on the surface of the bacterial lawn; each plaque ideally represents one active parent phage in the sample. The plaque count and dilution factor are then used to determine the active phage titer of the sample. Phage titers are therefore reported as plaqueforming units (PFUs) per mL. Because phage titers can vary over several orders of magnitude, change in phage titer is commonly reported as logarithmic change (base 10).

A subset of dry powder formulation studies on phages also determined the *in vitro* aerosol performance of the phage formulations and estimated the viable phage dose that could be delivered to the lungs. This is commonly referred to as the lung dose or fine particle dose/fraction (FPF) of the formulation. The lung phage dose of a dry powder formulation is a function of particle aerodynamic diameter, particle morphology, cohesive and adhesive particle forces (Ke et al., 2022), as well as the formulation’s capability to protect phages from any potential damage due to the shear and impact forces of powder dispersion and inhalation.

Some of the most important studies on phage formulations are summarized here, with a focus on the most important excipients for phage preservation and aerosol performance in dry inhalable powders (Table 2).

### 3.1 Spray drying

Chang et al. (2017) screened a series of excipients to stabilize phages during spray drying: trehalose, lactose, mannitol, glycine, leucine, PEG3000, and Pluronic F68. They employed three phages—PEV1, PEV20, and PEV61—that showed antimicrobial activity against 90 clinical and multidrug-resistant strains of *P. aeruginosa*. Trehalose and lactose were screened as the best excipients for preserving phage viability during spray drying. Then, different trehalose/lactose to leucine ratios were studied to determine the titer loss after spray drying. Higher sugar concentrations relative to leucine offered better protection. However, the highest sugar concentrations were not the best. The lowest titer loss was observed for all three phages with 20–5 mg/mL and 17–8 mg/mL sugar-leucine formulations for each sugar. Lactose provided better phage protection than trehalose, with titer loss of 0.3–0.4 and 0.5–0.9 logs, respectively. The leucine content in the formulation affected particle size and morphology. In formulations with high leucine concentrations, spherical particles of sizes less than 3 μm were obtained. Without leucine, particles formed large irregular agglomerates, which were unsuitable for inhalation. Aerosol performance analysis (MSLI) of the spray-dried formulations screened for high phage stability exhibited a fine particle fraction (FPF) greater than 50 %. The highest FPFs (65–67 %) were observed in formulations containing 0.0027 mg/mL Pluronic F68 (Chang et al., 2017).

It has been reported that leucine, which is usually included to improve powder dispersibility, can protect phages from thermal and desiccation stress during spray drying by forming a crystalline shell on the particle surface and displacing the phage particles to the core (Ke et al., 2022). It also offers moderate protection from moisture absorption during storage, which prevents crystallization of the amorphous sugar matrix (Li et al., 2016; 2017). However, leucine alone is a poor stabilizer of proteins in dry powders because it has a strong tendency to crystallize and cause phase separation of biologics from the protective matrix (Chen et al., 2021).

Spray dried powders containing phage cocktails were studied by Matinkhoo et al. (2011). The excipient matrix included trehalose (76 %), leucine (20 %), and optionally a third excipient (2 % of Pluronic surfactant, tyloxapol surfactant or casein Na salt). Casein was tested as a potential thermal stabilizer of phages based on the protective effect of milk on phages. The two surfactants were added to aid phage dispersion in the feed suspension for spray drying. Different phages showed varying losses from the spray drying process; but all phages in general experienced approximately 0.5 log losses. To measure the delivered lung dose, powder was dispersed through an Aerosolizer^®^ inhaler and an Alberta Idealized throat. The trehalose-leucine-casein matrix with a mass ratio of 0.76:0.19:0.02 provided the most consistent delivery of powder mass to the lungs (approx. 70 %) across all phages/cocktails (Matinkhoo et al., 2011).

Similarly, in a study by Leung et al. (2017), PEV2, a *Pseudomonas* phage, was spray dried into powder matrices with various compositions of trehalose (0–80 %), mannitol (0–80 %), and L-leucine (20 %). Trehalose content >40 % helped preserve phages with 1.3 log loss. At 0 % and 20 % trehalose, log titer losses were 2.4 and 5.1, respectively. Mannitol and leucine did not provide protection when used alone. The recovery and respirable fraction of phages were measured using an Osmohaler at 100 L/min for 2.4 s. All formulations had FPF values of 40–48 %. Low phage recovery was observed during aerosol testing (20–53 %), which was attributed to the inactivation of phages on the particle surface due to impaction with the inhaler and other surfaces (Leung et al., 2017).

These studies highlight the protective effects of sugars such as trehalose, lactose, and sucrose on proteins and other biological drug materials against the drying and thermal stresses of spray drying.

Another interesting excipient for spray-dried biological formulations is mannitol. Mannitol usually has a poor stabilizing effect because it tends to crystallize swiftly after spray drying and thus does not form an amorphous matrix with biologics. However, mannitol can be beneficial to stability when used in a smaller fraction with glassy sugars because it may help prevent protein aggregation and deamidation as long as mannitol does not crystallize in the solid matrix (Cleland et al., 2001; Leung et al., 2017; Yan et al., 2021).

Yan et al. (2021) studied the formulation of the anti-*Acinetobacter baumannii* Myoviridae phage AB406 as spray-dried powders consisting of three excipients of trehalose (40–80 %), mannitol (40–0 %), and L-leucine (20 %). Three total solid contents (20, 40 and 60 mg/mL) were assessed. The titer loss due to spray drying was found to be around 0.3–0.5 log for all compositions. Both trehalose-leucine and trehalose-mannitol-leucine systems were similar in their ability to stabilize the phage. In contrast to the study by Leung et al. (2017), the addition of 40 % mannitol did not reduce the survival of AB406 phages after spray drying. The formulations with mannitol had significantly more stable phage titers during storage at room temperature and <20 % relative humidity (RH) than trehalose-leucine only formulations (Yan et al., 2021). This result was attributed to the superior ability of mannitol to prevent protein aggregation and deamidation compared with disaccharides alone (Cleland et al., 2001). Contrary to the expectations of higher protein stabilization in a glassy matrix, the 40 % mannitol formulation with a low glass transition temperature (*T*_g_) (~15 °C) was more stable than the 80 % trehalose formulation (*T*_g_~110 °C), indicating that *T*_g_ alone may not be a good indicator of phage stability in dry powders. The aerosol performance of the formulations was measured at low humidity: formulations with higher mannitol content and lower total solid content showed higher FPF (Yan et al., 2021). The authors explain this trend to be due to the ability of mannitol to prevent particle merging under dry conditions and the slightly smaller particle sizes of the formulations with lower total solid content. However, exposure to 65 % moisture for 1 h before aerosol testing caused a composition-dependent reduction in FPF: the highest reduction was observed with 20 % mannitol (>30 %), followed by 40 % mannitol (15 %) and 0 % mannitol (~ 5 %). This was attributed to higher moisture uptake and recrystallization of mannitol in the formulation with 20 % mannitol (and 60 % trehalose), which could have led to the formation of strong agglomerates or aggregates (Arte et al., 2024).

In a study by Vandenheuvel et al. (2013), *Pseudomonas* phage LUZ19 and *Staphylococcus* phage Romulus were formulated using spray drying. Trehalose showed good phage stabilization, whereas lactose and dextran 35 exhibited poor phage stabilization. The authors concluded that the reducing property of lactose can damage the protein structure of phage particles and reduce their infectivity. Poor phage survival in the presence of Dextran 35 was related to insufficient H-bonding capacity of dextran to phage particles after dehydration (Vandenheuvel et al., 2013). The effects of two inlet temperatures (85 °C vs. 100 °C) and two atomization air flow rates (6 vs 12 L/min) on the formulation phage titer were tested. Lower inlet temperature and atomization flow rates favored phage viability. At identical spray drying parameters and excipient compositions, the Romulus phage experienced much higher titer loss than the LUZ19 phage. This could be attributed to the larger and more delicate structure of the former, which increased its susceptibility to the shear stress of spray drying (Vandenheuvel et al., 2013).

This group later studied the storage stabilities of spray-dried LUZ19 and Romulus phage formulations at two temperatures (4 °C and 25 °C) and two humidity conditions (0 % and 54 %RH) for 12 months (Vandenheuvel et al., 2014). Trehalose 4 % w/v was used as the stabilizer. At 54 %RH, regardless of temperature, the powders absorbed moisture and trehalose crystallized (forming trehalose dihydrate), resulting in the loss of phage viability over time. Storage at 25 °C caused thermal instability of the phages; phage loss was observed at both 0 % and 54 %RHs. At 0 % RH and 25 °C, phage loss occurred in the absence of trehalose crystallization.

In a study by LeClair et al. (2016), a human type 5 adenoviral vector (AdHu5) was formulated as a dry powder via spray drying to improve storage stability. Three excipient systems were tested: 100 % leucine, 90/10 lactose/trehalose, and 67/33 mannitol/dextran. The loss of viability of the viral vector due to spray drying were 2.6 ± 0.5 log for leucine, 0.7 ± 0.1 for trehalose/lactose, and 0.3 ± 0.1 log for mannitol/dextran. The authors explained this to be due to different degrees of encapsulation within the excipient matrix shell during droplet drying. The mannitol/dextran matrix encapsulated the virus particles better than other excipients because of the lower diffusion rate of dextran than disaccharides, which can cause it to precipitate at the surface (LeClair et al., 2016). The morphological analysis showed that the encapsulation of the virus was better in the trehalose/lactose matrix than the crystalline leucine matrix. The effects of temperature and ambient humidity on the storage stability of phages stored in a solid matrix were studied. The mannitol/dextran matrix provided the best thermal stability for the vector, showing 0.7 log loss at 20 °C and 10 %RH after 90 days, followed by trehalose/lactose with 3.1 log loss and leucine with 4.0 log loss. The lower phage stability observed in the lactose/trehalose matrix than in the mannitol/dextran matrix was attributed to the mobile amorphous state of the lactose/trehalose matrix. When the formulations were exposed to 45 %RH, all three formulations showed dramatically higher titer losses, which was attributed to moisture absorption and increased matrix mobility (LeClair et al., 2016). This is in contrast with the poor stabilizing properties of dextran and mannitol reported in other studies (Leung et al., 2017; Vandenheuvel et al., 2013). This result indicates that virus identity and manufacturing conditions strongly affect the stabilizing potential of excipient combinations.

Carrigy et al. (2019) studied the effects of the atomizing method and the type of spray dryer on the formulation of *Myoviridae* bacteriophages CP30A and CP20 that target *Campylobacter jejuni*. Trehalose (22.5 mg/mL) and leucine (7.5 mg/mL) were used as excipient matrix to stabilize the phage. The twin-fluid atomizer caused less phage inactivation (0.4 log_10_) than the vibrating mesh atomizer (0.8 log_10_). The study found that desiccation was more detrimental to phage viability than atomization (for both mesh nebulization and two-fluid atomizer): ~ 2.0 log loss was attributed to the drying process alone (Carrigy et al., 2019). This loss was quantitatively equivalent to room-temperature air drying loss (for 48 h), indicating high phage sensitivity to desiccation stress. The study also compared the titer loss between two spray dryers, B-90 and B-191. B-90, which does not have a cyclone for particle collection and uses electrostatic separation technology, showed a higher loss of phages than B-191. The authors inferred that cyclone separation of dry particles caused less damage to phage viability than electrostatic separation.

Another study by this group investigated the effect of trileucine or pullulan in comparison with leucine in combination with trehalose on phage viability loss due to spray drying (Carrigy et al., 2020). Pullalan (1.0 log PFU reduction) exhibited significantly less viability loss than the commonly used crystalline shell former leucine (1.7 log) when used in combination with trehalose (20–100 mg/mL). Trileucine-trehalose (4–100 mg/mL) combination showed excellent phage stabilization: overall, 0.6 log PFU loss was observed after spray drying and 1 month of dry storage at room temperature. The authors attribute the phage stabilization of trileucine and pullulan to their ability to form amorphous shells around dried particles during spray drying, which can protect phages from direct exposure to desiccation stress. On the other hand, leucine-trehalose formulation showed leucine crystallization after spray drying and a higher titer loss than trileucine and pullulan. This phage loss is likely due to destabilization associated with phase separation from the crystalline leucine and possible breakage by the growing crystals. Compared with the combination, trehalose and pullulan alone resulted in a higher phage loss of 2.4 logs during spray drying.

### 3.2 Lyophilization and spray freeze drying

*S. aureus* phage (ISP) was developed into solids using lyophilization (Merabishvili et al., 2013). The effects of different excipients and their concentrations on phage stability during lyophilization and storage were studied. Trehalose and sucrose were found to be the best stabilizers among the different excipients studied. The smallest phage losses after lyophilization were observed with 0.8 and 1 M sucrose, showing only 0.4–0.5 log loss. Activity reduction over 27 months of storage at 4 °C for all trehalose and sucrose formulations was within 1 log, except for 0.3 M trehalose. Phages suspended in Luria Bertani broth and 0.9 % saline without excipients and stored at 4 °C were tested as controls to study the storage stability of solid lyophilized formulations (Merabishvili et al., 2013). ISP was stable in LB showing only 1 log loss after 21 months. However, phage loss occurred steadily in the saline: 1 log loss after 12 months and, a further 1 log loss after 21 months. Glycine provided minimal protection when used as the sole excipient because it crystallized during lyophilization. After lyophilization with 0.1 or 0.5 M mannitol, high loss of phages (8 and 4 logs, respectively) was observed. This result was attributed to the crystallization of mannitol.

Polymers may stabilize lyophilized proteins due to their preferential exclusion, surface activity, steric hindrance to protein interaction, and increased solution viscosity (Wang, 2000). However, 1 % and 5 % PVP solutions showed complete phage inactivation before lyophilization, as reported by Merabishvili et al. (2013). Lyophilization with polyhydric polymeric solvent PEG 6000 at 1 % and 5 % showed log titer losses of 1.8 and 5.0, respectively. The authors also found that the type of phage significantly affects their survival after drying, and processing conditions are not the sole determinants of phage stability. Therefore, lyophilization conditions must be optimized for each individual phage (Merabishvili et al., 2013).

Golshahi et al. (2011) prepared inhalable phage formulations via lyophilization followed by milling. Bacteriophages KS4-M and ΦKZ were lyophilized in a 60–40 w/w lactose-lactoferrin matrix and milled in a mixer mill (without beads) for 5 min to generate respirable powders. Lactoferrins are antimicrobial peptides naturally found in the secretory fluids of mammals. The combination of lactoferrin and phages for treating persistent bacterial infections has been a topic of interest (Kosznik-Kwaśnicka et al., 2022; Zarzosa-Moreno et al., 2020; Zimecki et al., 2008). No significant loss of viability was observed for either phage after lyophilization-milling processing (Golshahi et al., 2011). The mass median aerodynamic diameter and geometric standard deviation of the powder were 3.4 and 1.9 μm as measured using Anderson Cascade Impactor.

A novel technique called atmospheric spray freeze drying was used to prepare dry powders of D29 phages with trehalose and mannitol (Ly et al., 2019). The process involved two steps: (i) spray freezing of the phage-excipient solution/suspension in a chamber cooled to −130 °C using liquid nitrogen, and (ii) atmospheric drying of the frozen solution by streaming progressively warmer air through the chamber, starting with a 2 h hold at −20 °C. The phage titer losses were 0.8 log for 7:3 trehalose-mannitol, 3.0 log for 1:1 trehalose-mannitol, and 1.5 log for 1:0 trehalose-mannitol (Ly et al., 2019). The presence of a lower concentration of mannitol appears to improve phage protection in the matrix; however, a higher fraction of mannitol in the matrix may lead to significant mannitol crystallization, leading to phase separation of the phages from the matrix.

In a study by Leung et al. (2016), the *Pseudomonas* PEV2 phage was formulated using two manufacturing technologies: spray drying (SD) and spray freeze drying (SFD). The study tested two formulation compositions: 60-20-20 (F1) and 40-40-20 (F2) trehalose-mannitol-leucine. Phage titer loss and *in vitro* aerosol performance were measured after preparation. The effect of manufacturing methods on phage activity was also examined. Phage loss during droplet generation was higher with the ultrasonic nozzle (used for SFD) than the 2-fluid nozzle (used for SD) (2 log vs 0.75 log). However, the evaporative drying step of SD caused greater damage to the phage titer than the freeze-drying step of SFD (excluding the spraying loss). It is interesting to note that overall, phage titer loss was lower for spray-dried formulations than for spray freeze-dried formulations in this study (Leung et al., 2016). When different compositions were considered, SFD-F1 exhibited better phage preservation than SFD-F2. This result was attributed to the higher amount of trehalose. However, SD-F1 and SD-F2 did not differ in terms of overall phage titer loss. The recovery of phages during *in vitro* aerosol testing varied substantially between SD and SFD (20 % vs 80 %) (Leung et al., 2016). Nonetheless, higher total lung phage doses (in PFU) were obtained for SD than for SFD.

## 4. Current challenges in phage therapy

Phages are an effective treatment option for difficult bacterial infections because of their specificity and adaptability. However, phage therapy has certain drawbacks and faces important hurdles: (1) Despite some animal studies and clinical cases that support the efficacy and safety of phages, they are a unique and novel class of drugs for which the regulatory pathway of development is yet to be paved (Kortright et al., 2019). (2) Phages need to be selected specifically for a patient’s needs because phages have a narrow host range. This can limit the accessibility of phage treatments and complicate the design of phage products for broader applications (Bhadoriya et al., 2023). It is also critical to select a phage that obligately infects and kills bacteria. (3) The FDA requires phages to be thoroughly evaluated for possible safety concerns. For instance, phages should not contain genes that express metabolites toxic to humans (Kortright et al., 2019), and they should not be able to transfer genes between bacteria (Doub, 2021). Finally, it is important to assess the risk of endotoxin release from lysed bacterial cells during phage product preparation and during its action in humans (Kortright et al., 2019). (4) Research on the interaction of phages with the human immune system is still in its infancy (Loc-Carrillo and Abedon, 2011; Roach et al., 2017). (5) Due to the omnipresence of phages in the environment, humans may possess neutralizing antibodies. Thus, therapeutic phages may be rendered ineffective by human immune mechanisms (Kortright et al., 2019). However, a recent article by Roach et al. (2017) showed that the innate immune response is important for the treatment of respiratory bacterial infection using phages. The results also highlight the ability of the host innate immune response to kill phage-resistant subpopulations of bacteria if a substantial fraction of the bacteria is phage-sensitive. Moreover, phages were well tolerated by the subject and were not neutralized by the immune system (Roach et al., 2017). (6) Phages are deemed unfit to target intracellular bacteria such as *Mycobacterium tuberculosis* (Kortright et al., 2019). (7) Bacteria can inevitably develop mechanisms to evade a specific phage, and in turn, the phage would need to be modified to target resistant bacteria. Therefore, it is important to employ strategies such as phage cocktails (Gordillo Altamirano and Barr, 2021) and phage-antibiotic combinations (Tagliaferri et al., 2019) that can delay resistance development and improve bacterial killing.

There are currently important challenges associated with the formulation of phages as dry powders: (1) Based on the review of reported literature on phage formulation, each phage is unique in its stability properties in the solid state; thus, its manufacturing and formulation must be optimized on a case-by-case basis. This can make developing dry powders cumbersome for phages. Due to the relative ease of formulation, most clinical applications of phages employ nebulization (Chang et al., 2018); (2) There is limited understanding of optimal phage dose, dose frequency, administration timing, and the best combinations (phage-phage or phage-antibiotics) for a plethora of indications. Specifically, the optimal phage therapy for treating polymicrobial or chronic infections has yet to be thoroughly investigated in animal or human subjects; (3) Plaque assay is a critical test for quantifying active phages in a formulation. However, due to its inherent variability, it is difficult to quantify small reductions in phage titers due to destabilization associated with shelf storage (Bodier-Montagutelli et al., 2017). This can make long-term storage stability data ambiguous. The variability of this assay also affects the accuracy of viable phages in the fine particle fraction of a dry powder, which is an important indicator of the efficiency of lung delivery; (4) The correlation between real-time and accelerated storage stability of phages is not well established, so storage stability assessment of formulations requires long durations of time; (5) Finally, the characterization of phage cocktails or phage-antibiotic combinations can be complex. It is challenging to differentiate phages and determine their individual stability in a phage cocktail using plaque assays. The antibiotic in phage-antibiotic combination formulations can wipe out the host bacteria used for the plaque assay before the phage can infect, which can interfere with plaque formation and affect phage detection.

## 5. Conclusion

Bacteriophages are important alternative antimicrobial agents against multidrug-resistant pathogens. Although phages have a long path to regulatory approval as therapeutic agents, they hold great promise as adjuvants to antibiotics. Therefore, it is important to determine formulation strategies that preserve bacteriophage activity and stabilize them to achieve a reasonable shelf-life. Inhalable dry powders could be a viable treatment option for pulmonary infections. The dry state of the formulation better preserves biological activity than solutions or suspensions. This review summarized some important findings on the formulation of bacteriophages as inhaled dry powders.

The formulation of phages as dry powders with different excipients and processing conditions resulted in different outcomes in previous studies. Some general trends are observed in these studies, such as the prominence of trehalose, lactose, and sucrose as lyoprotectants compared with other excipients, such as mannitol, leucine, glycine, and polymers. Mannitol may not be a good stabilizer on its own, but its tendency to crystallize after drying may improve the particle structural integrity. Leucine is widely used in dry powder formulations to improve aerosol performance. Because the amphiphilic nature of leucine leads to its mobilization to the droplet surface during spray drying, it may reduce the accumulation of phage units on the particle surface. Phages present on the surface can be deactivated by thermal, desiccation, and mechanical stresses; thus, leucine may reduce phage inactivation during spray drying and dose delivery (dispersion). It also prevents moisture absorption by forming a crystalline and non-hygroscopic shell over the particle. In addition to these general trends, the effects of excipient composition and drying process on the viability loss of phages appeared to depend on the type of phage. Screening of excipients and optimization of processing conditions to improve phage viability in dry powder must be performed for each phage product individually. Overall, emerging phage therapies are still in their infancy; more fundamental and clinical studies are warranted to make full use of their therapeutic potential in tackling the global healthcare crisis of antibiotic resistance.

## Figures and Tables

**Table 1:** Bacteriophage products and their status of clinical evaluation as of May 5, 2024.

Product name	Company	Target pathogen	Site of infection and comorbidity	Mode of delivery	Development stage	Trial ID
BX004	BiomX	*P. aeruginosa*	Lungs with cystic fibrosis	Not known	Part 2 of Phase 1b/2a was completed successfully	NCT05498363
BX211	BiomX	*S. aureus*	Diabetic foot osteomyelitis (DFO)	Not known	Ongoing phase 2 study	
Tailwind (AP-PA02)	Armata Pharmaceuticals	*P. aeruginosa*	Noncystic fibrotic bronchiectasis	Inhaled	Recruiting patients for phase 2	NCT05616221
SWARM P.a. (AP-PA02)	Armata Pharmaceuticals	*P. aeruginosa*	Lung with cystic fibrosis	Inhaled	Phase 1b/2a study has been completed	NCT04596319
AP-SA02	Armata Pharmaceuticals	*S. aureus*	Bacteremia	Not known (Adjunct to antibiotic therapy)	Recruiting patients for phase 1b/2a	NCT05184764

**Table 2:** Summary of studies on the formulation of bacteriophage dry powders.

Study	Phage studied	Manufacturing method	Excipients studied	Best formulation for lyoprotection (lowest phage log_10_ loss)	Best formulation for storage (lowest phage log_10_ loss)	Notable observations and inferences
(Matinkhoo et al., 2011)	*Burkholderia cepacia* KS4-M and KS14 phages; *Pseudomonas* phage ΦKZ/D3	Spray drying	76:20 trehalose-leucine 3^rd^ excipient: Pluronic surfactant, tyloxapol surfactant, or casein Na salt	No significant differences among the tested compositions (~0.5)	—	Trehalose-leucine-casein (0.76–0.19–0.02) provided the most consistent *in vitro* delivery of powder mass to the lungs (Alberta Idealized Throat).
(Chang et al., 2017)	*Pseudomonas* PEV1, PEV20, and PEV61	Spray drying	Trehalose, lactose, mannitol, glycine, leucine, PEG3000 and Pluronic F68	80:20/68:32 lactose-leucine (0.3–0.4) 80:20/68:32 trehalose-leucine (0.5–0.9)	—	92:8 sugar-leucine formulations were not the best stabilizers.
(Chang et al., 2020)	PEV20	Spray drying	80:20 and 50:50 lactose-leucine	Both excipient systems performed similarly	Storage at 5 °C/15 *%* relative humidity (RH) resulted in the lowest titer reduction after 250 days (0.4–0.6 log loss). Phage stability was lower at 5 °C/33 %RH and 25 °C/15 %RH (~1.1 log) and lowest at 25 °C/33 %RH (3–4 log).	A higher titer loss during storage was observed at temperature-RH conditions, resulting in a smaller difference between the formulation glass transition temperature and storage temperature.
(Li et al., 2021)	Cocktail of PEV1, PEV20 (myoviruses), and PEV2 (a podovirus)	Spray drying	80:20 lactose-leucine	Phage losses varied with the type of phage: PEV1, PEV2, and PEV20 exhibited titer losses of 0.1, 1.3, and 0.7 log.	—	Powder dispersion testing with a low-resistance Osmohaler (using NGI, 100 L/min) and a high-resistance Osmohaler (using MSLI, 60 L/min) showed fine particle fractions ~45% and ~63 %.
(Leung et al., 2017)	*Pseudomonas* PEV2	Spray drying	Trehalose (80–0 %) Mannitol (0–80 %) Leucine (20 %)	Trehalose content > 40 % (1.3)	—	*In vitro* aerosol analysis (MSLI) showed FPF 40–48 %. The low recovery of phages during MSLI testing (20–53 %) was attributed to inactivation due to impaction.
(Yan et al., 2021)	*Acinetobacter baumannii* AB406 phage	Spray drying	Trehalose (40–80 %) Mannitol (40–0 %) Leucine (20 %) Total solid of feed: 20, 40, and 60 mg/mL	All compositions exhibited similar phage loss (0.3–0.5)	Mannitol improved storage stability (room temperature, <20 % RH)	*In vitro* aerosol analysis (MSLI) at low RH showed that the FPF was better with higher mannitol content and lower feed total solid content. However, exposure to 65 % RH significantly reduced their FPF.
(Vandenheuvel et al., 2013)	*Pseudomonas* LUZ19 phage *Staphylococcus* phage Romulus	Spray drying	Trehalose Lactose Dextran 35	Trehalose	—	Lower spray-drying inlet temperature and atomization flow rate favored higher formulation phage viability. Romulus phages, owing to their larger and more delicate structure, were more susceptible to inactivation than LUZ19 during spray drying.
(Vandenheuvel et al., 2014)	*Pseudomonas* LUZ19 phage *Staphylococcus* phage Romulus	Spray drying	100 % trehalose	—	Better stability at 4 °C and 0 % RH	At 54 % RH, regardless of temperature, the powder absorbed moisture and trehalose crystallized. Storage at 25 °C caused thermal instability of the phages.
(LeClair et al., 2016)	Human type 5 adenoviral vector	Spray drying	100 % leucine 90:10 lactose/trehalose, 67:33 mannitol/dextran	67:33 mannitol/dextran (0.3)	67:33 mannitol/dextran (0.7 after 90 days at 20 °C and 10 %RH)	The mannitol/dextran matrix was the best stabilizer because it possibly encapsulated the virus particles better than other excipients. Surprisingly, trehalose/lactose did not stabilize the virus well during storage (3.1 log loss).
(Carrigy et al., 2019)	*Campylobacter jejuni* CP30A and CP20	Spray drying	Trehalose (22.5 mg/mL) Leucine (7.5 mg/mL)	(*compared atomizing method and the type of spray dryer*)		The twin-fluid atomizer caused less phage inactivation (0.4 log) than the vibrating mesh atomizer (0.8 log). Desiccation was a more harmful stressor than atomization. Buchi B-90 spray dryer showed a higher loss of phages than B-191 spray dryer.
(Carrigy et al., 2020)	*Campylobacter jejuni* CP30A	Spray drying	Trehalose (100 mg/mL) along with pullulan (20 mg/mL), trileucine (4 mg/mL), or leucine (20 mg/mL) as shell formers	Trileucine-trehalose & Pullulan-trehalose	Trileucine-trehalose (4:100 mg/mL) (overall 0.6 log loss after spray drying and 1-month room temperature storage)	Trileucine and pullulan formed amorphous shells around the dried particles during spray drying, which protected phages from direct exposure to desiccation stress.
(Merabishvili et al., 2013)	*Staphylococcus aureus* phages	Lyophilization	Sucrose (0.3–1.0 M) Trehalose (0.3–1.0 M) Mannitol Glycine Polyvinylpyrrolidone PEG 6000	Trehalose and sucrose Sucrose at 0.8 and 1.0 M exhibited the lowest loss (0.4–0.5)	Most trehalose and sucrose solutions showed < 1 log loss after 27 months at 4 °C.	Phage stability in trehalose- and sucrose-lyophilized formulations was comparable to that of Luria Bertani broth during storage at 4 °C.
(Golshahi et al., 2011)	*Burkholderia cepacia* phage KS4-M and *Pseudomonas* phage ΦKZ	Lyophilization and milling	60:40 lactose-lactoferrin	—	—	Powders were inhalable with a mass median aerodynamic diameter of 3.4 μm. For KS4-M, lung phage dose was 3.4 × 10^6^ from a 9.8 × 10^6^ PFU inhaler load; for phage ΦKZ, 9.8 × 10^6^ from al.9 × 10^7^ inhaler load.
(Ly et al., 2019)	*Mycobacterium tuberculosis* phage D29	Atmospheric spray freeze drying	70:30, 50:50, and 100:0 trehalose-mannitol	70:30 trehalose-mannitol (0.8)	—	Trehalose remained amorphous and mannitol crystallized in the powder.
(Leung et al., 2016)	*Pseudomonas* PEV2	Spray freeze drying (SFD) and Spray drying (SD) were compared	60:20:20 (F1) and 40:40:20 (F2) trehalose-mannitol-leucine	F1 and F2 were similar after spray drying. When spray freeze dried, F1 was better than F2.	—	Phage loss during droplet generation was higher with the ultrasonic nozzle (2.00) than with the 2-fluid nozzle (0.75). The drying step of SD was more harmful to phage titer than the drying step of SFD.
(Leung et al., 2018)	*Pseudomonas* PEV2 and PEV40	Spray drying	70:30 and 60:40 trehalose-leucine	The loss of phage titer did not differ between the two excipient combinations. After spray drying, the PEV2 and PEV40 titers decreased by 0.7 log and PEV40 titer decreased by 0.2 log.	Tested at 4 °C and 20 °C under vacuum over 12 months. Both PEV2 formulations were stable at 4 °C. At 20 °C, 70:30 trehalose-leucine exhibited better PEV2 stability than 60:40 trehalose-leucine.	MSLI dispersion tests showed high variability in the percentage recovery of phages. PEV40 formulations lost 0.50 log phages after storage regardless of temperature. The dispersion flow rate (100 vs. 60 L/min) and contact with the dispersion surfaces were ruled out as factors associated with low recovery.

## Nomenclature

CFU: Colony-forming units
FPF: Fine particle fraction
PFU: Plaque-forming units
RH: Relative humidity
SD: Spray drying
SFD: Spray freeze drying

## References

[R1] Abedon ST, Phage therapy of pulmonary infections, Bacteriophage, 5 (2015) e1020260. 10.1080/21597081.2015.1020260 26442188 PMC4422798

[R2] Ács N, Gambino M, Brøndsted L, Bacteriophage enumeration and detection methods, Frontiers in Microbiology, 11 (2020) 594868. 10.3389/fmicb.2020.594868 33193274 PMC7644846

[R3] Arte KS, Tower CW, Mutukuri TT, Chen Y, Patel SM, Munson EJ, Zhou Q, Understanding the impact of mannitol on physical stability and aerosolization of spray-dried protein powders for inhalation, International Journal of Pharmaceutics, 650 (2024) 123698. 10.1016/j.ijpharm.2023.123698 38081559 PMC10907098

[R4] Bartlett JG, Gilbert DN, Spellberg B, Seven ways to preserve the miracle of antibiotics, Clinical Infectious Diseases, 56 (2013) 1445–1450. 10.1093/cid/cit070 23403172

[R5] Bhadoriya P, Sharma R, Mehrotra R, Kaur S, Srivastava I, Jain M, Kaushik P, A review on re-emerging bacteriophage therapy in the era of XDR, Biocell, 47 (2023) 1915–1930. 10.32604/biocell.2023.029564

[R6] Bodier-Montagutelli E, Morello E, L’Hostis G, Guillon A, Dalloneau E, Respaud R, Pallaoro N, Blois H, Vecellio L, Gabard J, Heuzé-Vourc’h N, Inhaled phage therapy: a promising and challenging approach to treat bacterial respiratory infections, Expert Opinion on Drug Delivery, 14 (2017) 959–972. 10.1080/17425247.2017.1252329 27776446

[R7] Cao F, Wang X, Wang L, Li Z, Che J, Wang L, Li X, Cao Z, Zhang J, Jin L, Xu Y, Evaluation of the efficacy of a bacteriophage in the treatment of pneumonia induced by multidrug resistance Klebsiella pneumoniae in mice, BioMed Research International, 1 (2015) 752930. 10.1155/2015/752930 PMC4387947 25879036

[R8] Carrigy NB, Liang L, Wang H, Kariuki S, Nagel TE, Connerton IF, Vehring R, Spray-dried anti-*Campylobacter* bacteriophage CP30A powder suitable for global distribution without cold chain infrastructure, International journal of pharmaceutics, 569 (2019) 118601. 10.1016/j.ijpharm.2019.118601 31394183

[R9] Carrigy NB, Liang L, Wang H, Kariuki S, Nagel TE, Connerton IF, Vehring R, Trileucine and pullulan improve anti-campylobacter bacteriophage stability in engineered spray-dried microparticles, Annals of Biomedical Engineering, 48 (2020) 1169–1180. 10.1007/s10439-019-02435-6 31845128

[R10] Chan BK, Turner PE, Kim S, Mojibian HR, Elefteriades JA, Narayan D, Phage treatment of an aortic graft infected with Pseudomonas aeruginosa, Evolution, Medicine, and Public Health, 2018 (2018) 60–66. 10.1093/emph/eoy005 29588855 PMC5842392

[R11] Chang L, Pikal MJ, Mechanisms of protein stabilization in the solid state, Journal of Pharmaceutical Sciences, 98 (2009) 2886–2908. 10.1002/jps.21825 19569054

[R12] Chang RY, Wong J, Mathai A, Morales S, Kutter E, Britton W, Li J, Chan H-K, Production of highly stable spray dried phage formulations for treatment of *Pseudomonas aeruginosa* lung infection, European Journal of Pharmaceutics and Biopharmaceutics, 121 (2017) 1–13. 10.1016/j.ejpb.2017.09.002 28890220 PMC5650506

[R13] Chang RYK, Chen K, Wang J, Wallin M, Britton W, Morales S, Kutter E, Li J, Chan H-K, Proof-of-principle study in a murine lung infection model of antipseudomonal activity of phage PEV20 in a dry-powder formulation, Antimicrobial Agents and Chemotherapy, 62 (2018). 10.1128/aac.01714-17 PMC5786808 29158280

[R14] Chang RYK, Kwok PCL, Khanal D, Morales S, Kutter E, Li J, Chan H-K, Inhalable bacteriophage powders: glass transition temperature and bioactivity stabilization, Bioengineering & Translational Medicine, 5 (2020) e10159. 10.1002/btm2.10159 32440564 PMC7237144

[R15] Chen Y, Ling J, Li M, Su Y, Arte KS, Mutukuri TT, Taylor LS, Munson EJ, Topp EM, Zhou QT, Understanding the impact of protein–excipient interactions on physical stability of spray-dried protein solids, Molecular Pharmaceutics, 18 (2021) 2657–2668. 10.1021/acs.molpharmaceut.1c00189 34096731 PMC10042268

[R16] Cleland JL, Lam X, Kendrick B, Yang J, Yang T.h., Overcashier D, Brooks D, Hsu C, Carpenter JF, A specific molar ratio of stabilizer to protein is required for storage stability of a lyophilized monoclonal antibody, Journal of Pharmaceutical Sciences, 90 (2001) 310–321. 10.1002/1520-6017(200103)90:3<310::AIDJPS6>3.0.CO;2-R 11170024

[R17] Comeau AM, Tétart F, Trojet SN, Prère M-F, Krisch HM, Phage-antibiotic synergy (PAS): β-lactam and quinolone antibiotics stimulate virulent phage growth, PLOS ONE, 2 (2007) e799. 10.1371/journal.pone.0000799 17726529 PMC1949050

[R18] Debarbieux L, Leduc D, Maura D, Morello E, Criscuolo A, Grossi O, Balloy V, Touqui L, Bacteriophages can treat and prevent pseudomonas aeruginosa lung infections, The Journal of Infectious Diseases, 201 (2010) 1096–1104. 10.1086/651135 20196657

[R19] Doub J, Risk of bacteriophage therapeutics to transfer genetic material and contain contaminants beyond endotoxins with clinically relevant mitigation strategies, Infection and Drug Resistance, 14 (2021) 5629–5637. 10.2147/IDR.S341265 34992389 PMC8711558

[R20] Golshahi L, Lynch KH, Dennis JJ, Finlay WH, In vitro lung delivery of bacteriophages KS4-M and ΦKZ using dry powder inhalers for treatment of Burkholderia cepacia complex and Pseudomonas aeruginosa infections in cystic fibrosis, Journal of Applied Microbiology, 110 (2011) 106–117. 10.1111/j.1365-2672.2010.04863.x 20875034

[R21] Gordillo Altamirano FL, Barr JJ, Unlocking the next generation of phage therapy: the key is in the receptors, Current Opinion in Bio-technology, 68 (2021) 115–123. 10.1016/j.copbio.2020.10.002 33202354

[R22] Gould IM, Bal AM, New antibiotic agents in the pipeline and how they can help overcome microbial resistance, Virulence, 4 (2013) 185–191. 10.4161/viru.22507 23302792 PMC3654619

[R23] Grasmeijer N, Stankovic M, de Waard H, Frijlink HW, Hinrichs WL, Unraveling protein stabilization mechanisms: vitrification and water replacement in a glass transition temperature controlled system, Biochim Biophys Acta, 1834 (2013) 763–769. 10.1016/j.bbapap.2013.01.020 23360765

[R24] Hauser AR, Jain M, Bar-Meir M, McColley SA, Clinical significance of microbial infection and adaptation in cystic fibrosis, Clinical Microbiology Reviews, 24 (2011) 29–70. 10.1128/cmr.00036-10 21233507 PMC3021203

[R25] Hoppentocht M, Dry powder inhalation of antibiotics: a promising approach for treatment of infectious diseases, doctoral thesis, University of Groningen, 2016, ISBN: 978-94-90791-44-5.

[R26] Hoyle N, Zhvaniya P, Balarjishvili N, Bolkvadze D, Nadareishvili L, Nizharadze D, Wittmann J, Rohde C, Kutateladze M, Phage therapy against Achromobacter xylosoxidans lung infection in a patient with cystic fibrosis: a case report, Research in Microbiology, 169 (2018) 540–542. 10.1016/j.resmic.2018.05.001 29777836

[R27] Kamal F, Dennis JJ, Burkholderia cepacia complex phage-antibiotic synergy (PAS): antibiotics stimulate lytic phage activity, Applied and Environmental Microbiology, 81 (2015) 1132–1138. 10.1128/AEM.02850-14 25452284 PMC4292504

[R28] Ke W-R, Chang RYK, Chan H-K, Engineering the right formulation for enhanced drug delivery, Advanced Drug Delivery Reviews, 191 (2022) 114561. 10.1016/j.addr.2022.114561 36191861

[R29] Kortright KE, Chan BK, Koff JL, Turner PE, Phage therapy: a renewed approach to combat antibiotic-resistant bacteria, Cell Host & Microbe, 25 (2019) 219–232. 10.1016/j.chom.2019.01.014 30763536

[R30] Kosznik-Kwaśnicka K, Kaźmierczak N, Piechowicz L, Activity of phage–lactoferrin mixture against multi drug resistant staphylococcus aureus biofilms, Antibiotics, 11 (2022) 1256. 10.3390/antibiotics11091256 36140035 PMC9495459

[R31] Kwok PCL, Chan H-K, Delivery of inhalation drugs to children for asthma and other respiratory diseases, Advanced Drug Delivery Reviews, 73 (2014) 83–88. 10.1016/j.addr.2013.11.007 24270011

[R32] LeClair DA, Cranston ED, Xing Z, Thompson MR, Evaluation of excipients for enhanced thermal stabilization of a human type 5 adenoviral vector through spray drying, International Journal of Pharmaceutics, 506 (2016) 289–301. 10.1016/j.ijpharm.2016.04.067 27130366

[R33] Leung SSY, Parumasivam T, Gao FG, Carrigy NB, Vehring R, Finlay WH, Morales S, Britton WJ, Kutter E, Chan H-K, Production of inhalation phage powders using spray freeze drying and spray drying techniques for treatment of respiratory infections, Pharmaceutical Research, 33 (2016) 1486–1496. 10.1007/s11095-016-1892-6 26928668 PMC5083036

[R34] Leung SSY, Parumasivam T, Gao FG, Carter EA, Carrigy NB, Vehring R, Finlay WH, Morales S, Britton WJ, Kutter E, Chan H-K, Effects of storage conditions on the stability of spray dried, inhalable bacteriophage powders, International Journal of Pharmaceutics, 521 (2017) 141–149. 10.1016/j.ijpharm.2017.01.060 28163231 PMC5389863

[R35] Leung SSY, Parumasivam T, Nguyen A, Gengenbach T, Carter EA, Carrigy NB, Wang H, Vehring R, Finlay WH, Morales S, Britton WJ, Kutter E, Chan H-K, Effect of storage temperature on the stability of spray dried bacteriophage powders, European Journal of Pharmaceutics and Biopharmaceutics, 127 (2018) 213–222. 10.1016/j.ejpb.2018.02.033 29486303 PMC5948144

[R36] Li L, Leung SSY, Gengenbach T, Yu J, Gao G, Tang P, Zhou Q, Chan H-K, Investigation of L-leucine in reducing the moisture-induced deterioration of spray-dried salbutamol sulfate power for inhalation, International Journal of Pharmaceutics, 530 (2017) 30–39. 10.1016/j.ijpharm.2017.07.033 28709940

[R37] Li L, Sun S, Parumasivam T, Denman JA, Gengenbach T, Tang P, Mao S, Chan H-K, l-Leucine as an excipient against moisture on in vitro aerosolization performances of highly hygroscopic spray-dried powders, European Journal of Pharmaceutics and Biopharmaceutics, 102 (2016) 132–141. 10.1016/j.ejpb.2016.02.010 26970252

[R38] Li M, Chang RYK, Lin Y, Morales S, Kutter E, Chan H-K, Phage cocktail powder for Pseudomonas aeruginosa respiratory infections, International Journal of Pharmaceutics, 596 (2021) 120200. 10.1016/j.ijpharm.2021.120200 33486032 PMC7904610

[R39] Livermore DM, Multiple mechanisms of antimicrobial resistance in pseudomonas aeruginosa: our worst nightmare?, Clinical Infectious Diseases, 34 (2002) 634–640. 10.1086/338782 11823954

[R40] Livermore DM, The threat from the pink corner, Annals of Medicine, 35 (2003) 226–234. 10.1080/07853890310001609 12846264

[R41] Loc-Carrillo C, Abedon ST, Pros and cons of phage therapy, Bacteriophage, 1 (2011) 111–114. 10.4161/bact.1.2.14590 22334867 PMC3278648

[R42] Ly A, Carrigy NB, Wang H, Harrison M, Sauvageau D, Martin AR, Vehring R, Finlay WH, Atmospheric spray freeze drying of sugar solution with phage D29, Frontiers in Microbiology, 10 (2019) 488. 10.3389/fmicb.2019.00488 30949139 PMC6436606

[R43] Matinkhoo S, Lynch KH, Dennis JJ, Finlay WH, Vehring R, Spray-dried respirable powders containing bacteriophages for the treatment of pulmonary infections, Journal of Pharmaceutical Sciences, 100 (2011) 5197–5205. 10.1002/jps.22715 22020816

[R44] Merabishvili M, Vervaet C, Pirnay J-P, De Vos D, Verbeken G, Mast J, Chanishvili N, Vaneechoutte M, Stability of staphylococcus aureus phage ISP after freeze-drying (lyophilization), PLOS ONE, 8 (2013) e68797. 10.1371/journal.pone.0068797 23844241 PMC3699554

[R45] Patton JS, Byron PR, Inhaling medicines: delivering drugs to the body through the lungs, Nature Reviews Drug Discovery, 6 (2007) 67–74. 10.1038/nrd2153 17195033

[R46] Roach DR, Leung CY, Henry M, Morello E, Singh D, Di Santo JP, Weitz JS, Debarbieux L, Synergy between the host immune system and bacteriophage is essential for successful phage therapy against an acute respiratory pathogen, Cell Host & Microbe, 22 (2017) 38–47.e34. 10.1016/j.chom.2017.06.018 28704651

[R47] Rossolini GM, Arena F, Pecile P, Pollini S, Update on the antibiotic resistance crisis, Current Opinion in Pharmacology, 18 (2014) 56–60. 10.1016/j.coph.2014.09.006 25254623

[R48] Ruest MK, Supina BSI, Dennis JJ, Bacteriophage steering of Burkholderia cenocepacia toward reduced virulence and increased antibiotic sensitivity, Journal of Bacteriology, 205 (2023) e00196–00123. 10.1128/jb.00196-23 37791751 PMC10601696

[R49] Ryan EM, Alkawareek MY, Donnelly RF, Gilmore BF, Synergistic phage-antibiotic combinations for the control of Escherichia coli biofilms in vitro, FEMS Immunology & Medical Microbiology, 65 (2012) 395–398. 10.1111/j.1574-695X.2012.00977.x 22524448

[R50] Santana H, Sotolongo J, González Y, Hernández G, Chinea G, Gerónimo H, Amarantes O, Beldarraín A, Páez R, Stabilization of a recombinant human epidermal growth factor parenteral formulation through freeze-drying, Biologicals, 42 (2014) 322–333. 10.1016/j.biologicals.2014.07.005 25190208

[R51] Semler DD, Goudie AD, Finlay WH, Dennis JJ, Aerosol phage therapy efficacy in Burkholderia cepacia complex respiratory infections, Antimicrobial Agents and Chemotherapy, 58 (2014) 4005–4013. 10.1128/aac.02388-13 24798268 PMC4068594

[R52] Scoffone VC, Barbieri G, Irudal S, Trespidi G, Buroni S, New antimicrobial strategies to treat multi-drug resistant infections caused by gram-negatives in cystic fibrosis, Antibiotics, 13 (2024) 71. 10.3390/antibiotics13010071 38247630 PMC10812592

[R53] Tagliaferri TL, Jansen M, Horz H-P, Fighting pathogenic bacteria on two fronts: phages and antibiotics as combined strategy, Frontiers in Cellular and Infection Microbiology, 9 (2019) 22. 10.3389/fcimb.2019.00022 30834237 PMC6387922

[R54] Torres-Barceló C, Hochberg ME, Evolutionary rationale for phages as complements of antibiotics, Trends in Microbiology, 24 (2016) 249–256. 10.1016/j.tim.2015.12.011 26786863

[R55] Uchiyama J, Shigehisa R, Nasukawa T, Mizukami K, Takemura-Uchiyama I, Ujihara T, Murakami H, Imanishi I, Nishifuji K, Sakaguchi M, Matsuzaki S, Piperacillin and ceftazidime produce the strongest synergistic phage–antibiotic effect in Pseudomonas aeruginosa, Archives of Virology, 163 (2018) 1941–1948. 10.1007/s00705-018-3811-0 29550930

[R56] Vandenheuvel D, Meeus J, Lavigne R, Van den Mooter G, Instability of bacteriophages in spray-dried trehalose powders is caused by crystallization of the matrix, International Journal of Pharmaceutics, 472 (2014) 202–205. 10.1016/j.ijpharm.2014.06.026 24950368

[R57] Vandenheuvel D, Singh A, Vandersteegen K, Klumpp J, Lavigne R, Van den Mooter G, Feasibility of spray drying bacteriophages into respirable powders to combat pulmonary bacterial infections, European Journal of Pharmaceutics and Biopharmaceutics, 84 (2013) 578–582. 10.1016/j.ejpb.2012.12.022 23353012

[R58] Ventola CL, The antibiotic resistance crisis: part 1: causes and threats, Pharmacy and Therapeutics, 40 (2015) 277–283. https://www.ncbi.nlm.nih.gov/pmc/articles/PMC4378521/25859123 PMC4378521

[R59] Walters RH, Bhatnagar B, Tchessalov S, Izutsu K-I, Tsumoto K, Ohtake S, Next generation drying technologies for pharmaceutical applications, Journal of Pharmaceutical Sciences, 103 (2014) 2673–2695. 10.1002/jps.23998 24916125

[R60] Wang W, Lyophilization and development of solid protein pharmaceuticals, International Journal of Pharmaceutics, 203 (2000) 1–60. 10.1016/S0378-5173(00)00423-3 10967427

[R61] Yan W, He R, Tang X, Tian B, Liu Y, Tong Y, To KKW, Leung SSY, The influence of formulation components and environmental humidity on spray-dried phage powders for treatment of respiratory infections caused by *Acinetobacter baumannii*, Pharmaceutics, 13 (2021) 1162. 10.3390/pharmaceutics13081162 34452123 PMC8401170

[R62] Zarzosa-Moreno D, Avalos-Gómez C, Ramírez-Texcalco LS, Torres-López E, Ramírez-Mondragón R, Hernández-Ramírez JO, Serrano-Luna J, de la Garza M, Lactoferrin and its derived peptides: an alternative for combating virulence mechanisms developed by pathogens, Molecules, 25 (2020) 5763. 10.3390/molecules25245763 33302377 PMC7762604

[R63] Zhou Q, Tang P, Leung SSY, Chan JGY, Chan H-K, Emerging inhalation aerosol devices and strategies: where are we headed?, Advanced Drug Delivery Reviews, 75 (2014) 3–17. 10.1016/j.addr.2014.03.006 24732364

[R64] Zimecki M, Artym J, Kocieba M, Weber-Dabrowska B, Lusiak-Szelachowska M, Górski A, The concerted action of lactoferrin and bacteriophages in the clearance of bacteria in sublethally infected mice, Postepy Hig Med Dosw (Online), 62 (2008) 42–46. https://pubmed.ncbi.nlm.nih.gov/18268472/18268472

